# Supplementary material for: A Likelihood Approach for Real-Time Calibration of Stochastic Compartmental Epidemic Models
Source: PLoS Comput Biol. 2017 Jan 17;13(1):e1005257. doi: 10.1371/journal.pcbi.1005257 (PMC5240920; doi:10.1371/journal.pcbi.1005257)
Supplement: S1 File — (TAR.GZ) [file pcbi.1005257.s014.tar.gz › HSPH_Online-SI-Revision/IPoi09/n100-mild/n100-mild_BENCH2_table0.pdf]

1	newly 3 weeks spec	2866.	4479.84	{2071., 8206.}	67.7251	0.207143	newly 3 weeks cumul	9776.	16 911.9	{9379., 25 366.}	76.9382	0.232769	newly 5 weeks spec	1628.	2447.08	{880., 5540.}	73.4466	0.217844	newly 5 weeks cumul	13 712.	22 743.9	{11 569., 37 391.}	72.5525	0.21965	newly 52 weeks cumul	16 805.	27 878.7	{13 081., 50 711.}	74.3219	0.21835
2	newly 3 weeks spec	3141.	5189.38	{2893., 8262.}	70.1281	0.214447	newly 3 weeks cumul	11 118.	19 610.8	{11 652., 28 008.}	78.8409	0.238774	newly 5 weeks spec	1875.	2944.72	{1402., 5018.}	67.2942	0.200643	newly 5 weeks cumul	15 610.	26 489.6	{16 077., 39 540.}	72.7699	0.223159	newly 51 weeks cumul	19 660.	33 840.3	{19 055., 51 804.}	76.1975	0.225829
3	newly 3 weeks spec	2615.	5583.05	{3036., 8265.}	114.441	0.302064	newly 3 weeks cumul	10 083.	22 381.3	{13 668., 29 791.}	121.971	0.328695	newly 5 weeks spec	1275.	2614.12	{1239., 4060.}	108.606	0.284562	newly 5 weeks cumul	13 206.	28 879.1	{16 826., 39 858.}	118.682	0.318025	newly 54 weeks cumul	16 122.	33 606.8	{18 989., 47 472.}	108.734	0.295181
4	newly 3 weeks spec	3002.	5138.41	{1900., 9654.}	84.7393	0.232918	newly 3 weeks cumul	14 624.	25 301.2	{11 979., 42 422.}	79.668	0.232937	newly 5 weeks spec	1149.	1649.66	{522., 3111.}	71.4488	0.222026	newly 5 weeks cumul	17 569.	29 924.3	{13 457., 51 171.}	78.4738	0.22808	newly 58 weeks cumul	18 847.	31 841.3	{14 018., 54 818.}	77.9458	0.226469
5	newly 3 weeks spec	2761.	4180.16	{1761., 6805.}	64.6931	0.205225	newly 3 weeks cumul	10 134.	18 252.6	{9273., 27 507.}	85.4387	0.253322	newly 5 weeks spec	1641.	1797.62	{730., 3067.}	48.916	0.199467	newly 5 weeks cumul	14 065.	22 832.2	{11 116., 35 017.}	70.5163	0.218898	newly 54 weeks cumul	17 712.	25 743.6	{12 104., 39 691.}	57.3494	0.188515
6	newly 3 weeks spec	2631.	5073.5	{3143., 7281.}	94.4736	0.263967	newly 3 weeks cumul	10 483.	19 949.	{14 071., 26 639.}	90.9666	0.265482	newly 5 weeks spec	1399.	2590.75	{1424., 4021.}	88.1094	0.239686	newly 5 weeks cumul	13 754.	26 199.5	{17 604., 36 063.}	91.415	0.262745	newly 52 weeks cumul	16 963.	31 469.6	{20 197., 44 215.}	86.9821	0.249822
7	newly 3 weeks spec	3036.	7826.6	{4118., 13 453.}	157.803	0.373139	newly 3 weeks cumul	13 981.	30 200.9	{20 528., 39 583.}	116.014	0.321702	newly 5 weeks spec	924.	3621.37	{1537., 6847.}	291.923	0.512673	newly 5 weeks cumul	16 613.	39 330.5	{24 607., 57 380.}	136.745	0.354444	newly 57 weeks cumul	17 582.	44 976.6	{26 837., 68 486.}	155.811	0.382073
8	newly 3 weeks spec	2036.	4933.84	{2409., 7867.}	143.103	0.350525	newly 3 weeks cumul	8038.	18 499.8	{10 574., 26 338.}	130.77	0.340776	newly 5 weeks spec	1057.	2844.71	{1172., 5332.}	170.316	0.368213	newly 5 weeks cumul	10 545.	25 160.4	{13 418., 37 584.}	139.078	0.352067	newly 51 weeks cumul	12 212.	32 482.7	{15 957., 52 826.}	166.391	0.387033
9	newly 3 weeks spec	3348.	7253.07	{3882., 12 594.}	118.738	0.30372	newly 3 weeks cumul	14 156.	28 143.5	{18 205., 40 435.}	100.149	0.283569	newly 5 weeks spec	1368.	3662.25	{1520., 7567.}	170.383	0.350213	newly 5 weeks cumul	17 721.	37 053.8	{23 053., 56 869.}	109.843	0.298826	newly 56 weeks cumul	20 118.	43 879.4	{25 493., 72 992.}	119.066	0.309201
10	newly 3 weeks spec	2716.	3535.3	{1619., 7074.}	58.768	0.204139	newly 3 weeks cumul	9804.	13 656.6	{7381., 22 046.}	54.8452	0.181452	newly 5 weeks spec	1557.	1986.14	{773., 4159.}	68.8757	0.239856	newly 5 weeks cumul	13 469.	18 323.5	{9222., 34 467.}	56.3722	0.18804	newly 50 weeks cumul	16 876.	22 981.7	{10 736., 44 314.}	60.161	0.197527
11	newly 3 weeks spec	3997.	9254.56	{7160., 11 163.}	131.731	0.357032	newly 3 weeks cumul	15 533.	28 877.3	{21 679., 33 194.}	90.0565	0.276865	newly 5 weeks spec	1963.	6869.87	{4484., 10 840.}	249.968	0.522047	newly 5 weeks cumul	20 137.	43 874.8	{36 301., 50 145.}	117.881	0.333363	newly 54 weeks cumul	22 930.	65 717.1	{50 208., 85 348.}	186.599	0.449124
12	newly 3 weeks spec	3071.	5761.4	{3353., 9228.}	92.5425	0.262801	newly 3 weeks cumul	10 922.	19 805.8	{13 616., 26 689.}	84.0305	0.254036	newly 5 weeks spec	2036.	3740.49	{1702., 7281.}	93.0433	0.245027	newly 5 weeks cumul	15 499.	28 283.9	{17 685., 41 506.}	86.3682	0.254526	newly 50 weeks cumul	21 968.	38 490.4	{21 555., 62 896.}	81.0315	0.234719
13	newly 3 weeks spec	3153.	6311.86	{3442., 9709.}	103.592	0.279621	newly 3 weeks cumul	12 554.	25 251.9	{16 667., 34 532.}	102.513	0.289394	newly 5 weeks spec	1478.	2873.06	{1397., 4724.}	100.269	0.262235	newly 5 weeks cumul	16 279.	32 498.	{20 281., 46 182.}	101.746	0.284218	newly 55 weeks cumul	18 456.	37 439.8	{22 472., 54 416.}	105.214	0.288349
14	newly 3 weeks spec	2684.	5553.09	{1965., 10 428.}	115.459	0.290261	newly 3 weeks cumul	12 513.	24 988.9	{11 076., 40 951.}	103.69	0.282143	newly 5 weeks spec	929.	2216.41	{596., 4080.}	149.356	0.308706	newly 5 weeks cumul	15 065.	30 779.5	{12 675., 52 528.}	109.162	0.288809	newly 57 weeks cumul	16 544.	33 927.7	{13 521., 58 093.}	110.619	0.287527
15	newly 3 weeks spec	3944.	9412.41	{6827., 11 291.}	138.651	0.369472	newly 3 weeks cumul	16 141.	28 979.9	{21 315., 33 464.}	80.5925	0.250734	newly 5 weeks spec	1651.	7063.	{3964., 11 242.}	327.801	0.603732	newly 5 weeks cumul	20 293.	44 440.9	{34 903., 51 029.}	118.996	0.335357	newly 54 weeks cumul	22 727.	67 555.9	{46 002., 88 345.}	197.249	0.461484
16	newly 3 weeks spec	2270.	4459.85	{2603., 7397.}	97.5008	0.264229	newly 3 weeks cumul	7578.	16 371.	{11 025., 23 214.}	116.034	0.316895	newly 5 weeks spec	1786.	2617.24	{1243., 4612.}	63.9322	0.191706	newly 5 weeks cumul	11 349.	22 469.	{14 139., 33 944.}	98.5474	0.274268	newly 50 weeks cumul	16 325.	28 882.8	{17 050., 46 151.}	78.9017	0.221902
17	newly 3 weeks spec	4099.	7686.9	{3488., 13 314.}	94.0234	0.259193	newly 3 weeks cumul	15 754.	28 433.8	{17 393., 37 620.}	82.6534	0.249615	newly 5 weeks spec	1851.	3772.54	{1304., 8126.}	114.921	0.280982	newly 5 weeks cumul	20 371.	37 776.	{20 827., 55 946.}	88.4747	0.256524	newly 56 weeks cumul	22 951.	44 114.3	{22 691., 70 695.}	95.643	0.266792
18	newly 3 weeks spec	3126.	6000.17	{2842., 10 109.}	95.7804	0.251752	newly 3 weeks cumul	17 638.	32 034.4	{19 566., 47 130.}	82.3232	0.240515	newly 5 weeks spec	665.	1571.	{717., 2691.}	138.028	0.316737	newly 5 weeks cumul	19 872.	36 741.5	{21 538., 55 145.}	85.8154	0.245442	newly 60 weeks cumul	20 225.	38 219.	{22 116., 57 600.}	89.7497	0.25292
19	newly 3 weeks spec	3015.	6665.19	{4428., 9212.}	122.126	0.329207	newly 3 weeks cumul	11 397.	21 767.8	{14 913., 26 622.}	91.3365	0.273252	newly 5 weeks spec	1523.	4737.64	{2367., 8628.}	212.073	0.442138	newly 5 weeks cumul	15 147.	32 266.9	{23 256., 41 638.}	113.49	0.316805	newly 51 weeks cumul	18 357.	46 213.9	{28 828., 68 789.}	152.234	0.375799
20	newly 3 weeks spec	3891.	8100.47	{3443., 15 289.}	113.163	0.278426	newly 3 weeks cumul	17 885.	35 403.2	{21 919., 48 517.}	97.9493	0.277967	newly 5 weeks spec	1246.	2503.84	{823., 5272.}	117.651	0.276522	newly 5 weeks cumul	21 430.	42 646.8	{24 426., 63 562.}	99.293	0.273991	newly 60 weeks cumul	22 520.	45 151.6	{25 254., 68 933.}	100.918	0.274576
21	newly 3 weeks spec	3638.	2130.54	{902., 3998.}	57.9326	0.38023	newly 3 weeks cumul	16 322.	15 896.5	{7510., 28 619.}	43.8397	0.197954	newly 5 weeks spec	1041.	373.043	{129., 735.}	70.1834	0.57665	newly 5 weeks cumul	19 258.	17 183.5	{8183., 31 159.}	43.4371	0.212581	newly 60 weeks cumul	20 284.	17 442.7	{8342., 31 676.}	43.4465	0.219672
22	newly 3 weeks spec	3362.	4381.41	{2096., 7112.}	53.8667	0.190062	newly 3 weeks cumul	10 760.	16 690.8	{9460., 25 558.}	62.9096	0.196665	newly 5 weeks spec	2166.	2419.32	{1018., 4085.}	54.2139	0.221758	newly 5 weeks cumul	15 662.	22 407.8	{11 966., 34 778.}	56.8032	0.185766	newly 50 weeks cumul	22 682.	27 914.4	{13 957., 44 492.}	48.4606	0.17949
23	newly 3 weeks spec	3951.	9546.18	{6402., 12 041.}	141.614	0.372533	newly 3 weeks cumul	16 287.	30 396.9	{21 181., 35 490.}	89.2477	0.272561	newly 5 weeks spec	1653.	6710.27	{3763., 11 172.}	305.945	0.5763	newly 5 weeks cumul	20 536.	45 310.	{33 862., 53 506.}	120.637	0.3378	newly 55 weeks cumul	22 473.	64 617.	{44 674., 85 657.}	187.532	0.446888
24	newly 3 weeks spec	2779.	5453.06	{1894., 10 223.}	106.592	0.26565	newly 3 weeks cumul	13 850.	27 613.1	{12 867., 44 788.}	102.196	0.275718	newly 5 weeks spec	702.	1527.52	{482., 2949.}	129.561	0.287647	newly 5 weeks cumul	16 098.	32 096.3	{14 399., 53 519.}	103.263	0.27415	newly 59 weeks cumul	16 529.	33 608.3	{14 894., 56 506.}	107.142	0.279751
25	newly 3 weeks spec	3462.	7355.42	{4949., 8937.}	116.797	0.340554	newly 3 weeks cumul	12 350.	22 561.2	{17 499., 26 088.}	88.261	0.290964	newly 5 weeks spec	2037.	5823.33	{3077., 8379.}	187.28	0.435192	newly 5 weeks cumul	17 226.	35 071.8	{25 893., 41 380.}	107.402	0.320725	newly 50 weeks cumul	22 743.	58 182.7	{38 388., 76 529.}	155.827	0.394079
26	newly 3 weeks spec	3048.	8074.31	{5544., 10 108.}	164.905	0.412091	newly 3 weeks cumul	12 655.	25 933.1	{20 886., 30 283.}	107.525	0.313154	newly 5 weeks spec	1843.	5933.15	{3489., 9393.}	221.929	0.482736	newly 5 weeks cumul	16 877.	38 902.	{28 287., 46 258.}	130.503	0.355563	newly 52 weeks cumul	20 285.	59 683.9	{40 816., 81 971.}	194.227	0.455244
27	newly 3 weeks spec	3519.	7360.67	{5461., 9065.}	111.338	0.319621	newly 3 weeks cumul	12 256.	22 385.7	{17 655., 26 231.}	87.4951	0.276093	newly 5 weeks spec	1833.	6097.78	{3705., 8766.}	232.666	0.500924	newly 5 weeks cumul	16 564.	35 298.1	{27 420., 41 509.}	114.351	0.325896	newly 50 weeks cumul	21 086.	61 563.9	{41 942., 82 624.}	191.966	0.451805
28	newly 3 weeks spec	2682.	3579.37	{1527., 7444.}	60.7108	0.203445	newly 3 weeks cumul	8965.	14 009.3	{6886., 24 881.}	65.9794	0.198278	newly 5 weeks spec	1792.	1823.47	{654., 4317.}	61.7657	0.274537	newly 5 weeks cumul	12 944.	18 427.3	{8495., 35 296.}	59.4041	0.18834	newly 51 weeks cumul	19 275.	22 120.3	{9855., 44 197.}	50.2374	0.195467
29	newly 3 weeks spec	2188.	4716.63	{2480., 8062.}	116.342	0.295246	newly 3 weeks cumul	8468.	18 315.4	{11 435., 27 209.}	116.289	0.31497	newly 5 weeks spec	1093.	2471.88	{1052., 4931.}	130.096	0.288103	newly 5 weeks cumul	11 210.	24 278.7	{14 077., 37 680.}	116.58	0.307387	newly 53 weeks cumul	14 641.	29 145.5	{15 865., 49 865.}	100.32	0.266212
30	newly 3 weeks spec	3476.	8749.47	{5901., 11 741.}	151.952	0.387948	newly 3 weeks cumul	15 376.	30 070.7	{23 394., 36 112.}	97.9256	0.294548	newly 5 weeks spec	1413.	5669.7	{3128., 9960.}	301.253	0.568321	newly 5 weeks cumul	19 115.	42 921.3	{31 367., 53 003.}	124.543	0.343228	newly 55 weeks cumul	20 923.	58 831.1	{39 147., 83 849.}	181.179	0.43514
31	newly 3 weeks spec	2766.	6566.23	{3700., 9635.}	137.391	0.353496	newly 3 weeks cumul	10 733.	23 236.2	{15 761., 28 477.}	118.646	0.332968	newly 5 weeks spec	1803.	3934.15	{2029., 6667.}	120.584	0.311593	newly 5 weeks cumul	14 870.	32 359.8	{20 333., 43 723.}	117.618	0.322765	newly 52 weeks cumul	18 960.	42 655.2	{26 432., 61 122.}	125.044	0.331572
32	newly 3 weeks spec	2860.	4546.63	{2165., 7591.}	69.1287	0.209959	newly 3 weeks cumul	9853.	17 101.7	{9329., 26 244.}	78.9083	0.236475	newly 5 weeks spec	1902.	2623.67	{1018., 6402.}	63.2546	0.20925	newly 5 weeks cumul	14 117.	23 213.5	{11 979., 36 578.}	71.113	0.215848	newly 50 weeks cumul	21 462.	30 056.7	{13 957., 58 459.}	55.7842	0.184243
33	newly 3 weeks spec	1762.	9655.33	{4233., 15 984.}	447.976	0.690942	newly 3 weeks cumul	12 781.	39 043.8	{24 102., 49 883.}	205.483	0.469676	newly 5 weeks spec	298.	3475.34	{1183., 6330.}	1066.22	0.96624	newly 5 weeks cumul	13 856.	48 542.5	{28 106., 66 401.}	250.335	0.525078	newly 60 weeks cumul	13 996.	52 471.5	{29 369., 74 123.}	274.903	0.550856
34	newly 3 weeks spec	2684.	8146.67	{5189., 12 647.}	203.527	0.457322	newly 3 weeks cumul	12 876.	30 134.9	{22 811., 40 094.}	136.148	0.365534	newly 5 weeks spec	799.	4639.46	{2406., 7384.}	480.658	0.702976	newly 5 weeks cumul	15 119.	41 003.7	{28 820., 56 620.}	171.206	0.419707	newly 56 weeks cumul	15 839.	51 565.	{34 918., 72 344.}	225.557	0.491716
35	newly 3 weeks spec	685.	9209.02	{5341., 13 328.}	1244.38	1.09528	newly 3 weeks cumul	10 337.	39 877.5	{29 213., 50 997.}	285.774	0.576365	newly 5 weeks spec	47.	3431.07	{1705., 4901.}	7200.14	1.79633	newly 5 weeks cumul	10 552.	49 083.1	{34 616., 65 849.}	365.154	0.653614	newly 60 weeks cumul	10 552.	53 507.8	{36 789., 72 163.}	407.087	0.688112
36	newly 3 weeks spec	2872.	4043.46	{1481., 9344.}	69.0536	0.226305	newly 3 weeks cumul	12 863.	23 826.5	{12 261., 41 268.}	90.4424	0.252962	newly 5 weeks spec	910.	858.829	{263., 2242.}	56.9981	0.277707	newly 5 weeks cumul	15 281.	26 582.3	{13 136., 48 283.}	81.6493	0.234127	newly 60 weeks cumul	16 833.	27 247.3	{13 307., 49 992.}	72.5252	0.217644
37	newly 3 weeks spec	3362.	8299.59	{6198., 11 021.}	148.771	0.388263	newly 3 weeks cumul	13 277.	26 892.9	{20 146., 31 919.}	106.272	0.316801	newly 5 weeks spec	1507.	5928.56	{3562., 10 202.}	293.401	0.563412	newly 5 weeks cumul	17 055.	39 958.	{31 584., 49 523.}	135.671	0.367024	newly 53 weeks cumul	19 554.	59 302.9	{42 134., 83 760.}	203.278	0.467519
38	newly 3 weeks spec	2972.	8544.45	{3767., 15 609.}	189.07	0.403562	newly 3 weeks cumul	16 469.	35 381.9	{23 492., 47 635.}	115.652	0.319806	newly 5 weeks spec	612.	2991.87	{863., 7970.}	389.904	0.543781	newly 5 weeks cumul	18 339.	43 704.3	{26 328., 63 202.}	139.151	0.356923	newly 60 weeks cumul	18 646.	46 794.	{27 115., 73 648.}	151.751	0.374462
39	newly 3 weeks spec	2387.	4392.05	{1577., 9781.}	98.1412	0.245582	newly 3 weeks cumul	10 499.	20 742.2	{9105., 37 990.}	102.246	0.267737	newly 5 weeks spec	937.	1488.02	{464., 3582.}	89.1086	0.245375	newly 5 weeks cumul	12 897.	24 840.6	{10 477., 47 450.}	98.7999	0.256121	newly 57 weeks cumul	14 107.	26 592.1	{10 969., 51 694.}	95.5988	0.248005
40	newly 3 weeks spec	2220.	5525.91	{2266., 11 804.}	150.606	0.321302	newly 3 weeks cumul	13 427.	27 413.2	{14 655., 48 908.}	105.132	0.275859	newly 5 weeks spec	616.	1819.94	{591., 3452.}	198.53	0.332811	newly 5 weeks cumul	15 217.	32 463.4	{16 397., 59 559.}	114.417	0.28752	newly 59 weeks cumul	15 737.	34 443.8	{16 959., 63 292.}	119.927	0.293876
41	newly 3 weeks spec	3803.	7759.35	{3352., 15 077.}	109.21	0.276013	newly 3 weeks cumul	18 778.	36 214.5	{21 098., 49 632.}	93.1623	0.26445	newly 5 weeks spec	894.	2479.76	{819., 4872.}	180.721	0.344991	newly 5 weeks cumul	21 558.	43 232.9	{23 649., 65 474.}	101.109	0.276791	newly 60 weeks cumul	22 565.	45 834.	{24 462., 72 958.}	103.852	0.279821
42	newly 3 weeks spec	3308.	6674.42	{3039., 10 606.}	104.852	0.276869	newly 3 weeks cumul	14 867.	28 390.4	{16 090., 39 725.}	91.8229	0.263943	newly 5 weeks spec	1090.	2702.98	{1109., 4389.}	149.876	0.337033	newly 5 weeks cumul	17 792.	35 457.4	{18 879., 51 345.}	100.251	0.27738	newly 57 weeks cumul	18 900.	39 456.4	{20 358., 57 774.}	109.647	0.293677
43	newly 3 weeks spec	4665.	3933.23	{1432., 8754.}	58.8921	0.311841	newly 3 weeks cumul	19 701.	23 799.8	{11 754., 43 578.}	49.0066	0.180397	newly 5 weeks spec	1372.	830.273	{255., 1809.}	62.394	0.429954	newly 5 weeks cumul	23 754.	26 480.9	{12 607., 50 094.}	49.2959	0.197355	newly 60 weeks cumul	25 089.	27 112.3	{12 792., 51 770.}	49.0008	0.202563
44	newly 3 weeks spec	3246.	8057.9	{4451., 13 778.}	148.491	0.354085	newly 3 weeks cumul	17 555.	34 266.6	{24 263., 46 828.}	97.6057	0.28712	newly 5 weeks spec	782.	3399.46	{1366., 7156.}	334.713	0.526853	newly 5 weeks cumul	19 996.	42 985.6	{28 569., 62 727.}	115.083	0.313871	newly 59 weeks cumul	20 762.	47 927.5	{30 280., 77 347.}	130.885	0.338546
45	newly 3 weeks spec	3565.	6310.02	{3226., 10 493.}	82.2957	0.234168	newly 3 weeks cumul	13 069.	24 875.4	{15 391., 34 420.}	91.6548	0.26611	newly 5 weeks spec	1772.	2910.3	{1291., 5474.}	77.7383	0.222906	newly 5 weeks cumul	17 480.	32 193.4	{18 752., 47 998.}	86.2793	0.249626	newly 55 weeks cumul	20 564.	37 157.9	{20 989., 57 642.}	83.5703	0.240631
46	newly 3 weeks spec	4749.	9970.64	{6889., 12 944.}	109.952	0.311241	newly 3 weeks cumul	18 136.	32 961.4	{27 018., 37 942.}	82.8362	0.258359	newly 5 weeks spec	1998.	6360.41	{3600., 10 640.}	218.339	0.470398	newly 5 weeks cumul	23 379.	47 529.6	{36 459., 56 780.}	103.3	0.302613	newly 56 weeks cumul	26 044.	63 712.7	{44 986., 85 566.}	144.635	0.377638
47	newly 3 weeks spec	3376.	9140.44	{6173., 11 884.}	170.748	0.418478	newly 3 weeks cumul	16 086.	32 214.2	{22 479., 38 919.}	100.402	0.292433	newly 5 weeks spec	1272.	5472.3	{2931., 7905.}	330.212	0.589742	newly 5 weeks cumul	19 497.	44 926.7	{33 134., 55 578.}	130.429	0.354044	newly 56 weeks cumul	20 865.	57 797.1	{39 280., 75 666.}	177.005	0.42761
48	newly 3 weeks spec	3671.	7992.23	{5594., 9567.}	121.535	0.343249	newly 3 weeks cumul	14 471.	24 736.9	{20 556., 28 124.}	78.7199	0.266681	newly 5 weeks spec	1686.	6289.55	{4350., 8422.}	273.046	0.558242	newly 5 weeks cumul	18 539.	38 253.6	{28 674., 43 914.}	109.175	0.317949	newly 51 weeks cumul	21 237.	65 207.1	{49 708., 82 896.}	207.045	0.478379
49	newly 3 weeks spec	2539.	5021.65	{2334., 8711.}	101.919	0.253904	newly 3 weeks cumul	11 374.	22 612.4	{13 252., 34 382.}	99.0267	0.27115	newly 5 weeks spec	806.	1937.79	{730., 3185.}	145.684	0.286534	newly 5 weeks cumul	13 685.	27 733.9	{15 424., 43 891.}	103.217	0.27271	newly 57 weeks cumul	14 608.	30 245.3	{16 302., 47 768.}	107.758	0.276839
50	newly 3 weeks spec	2519.	5772.63	{2157., 13 650.}	135.536	0.298286	newly 3 weeks cumul	12 653.	25 333.7	{13 168., 43 357.}	103.092	0.278845	newly 5 weeks spec	744.	2025.86	{594., 5297.}	180.951	0.322426	newly 5 weeks cumul	14 778.	30 924.9	{14 981., 58 528.}	112.661	0.28828	newly 58 weeks cumul	15 627.	33 282.6	{15 619., 64 893.}	116.574	0.291163
